# Death in Buffalo of Dr. Hernan P. Babcock, of Oakland, California

**Published:** 1879-02

**Authors:** 


### Death of Heman P. Babcock of Oakland, California.

In our last JOURNAL we noticed the death of Dr. Heman P. Babcock, which occurred in this city Dec. 27, 1878. Prof. T. F. Rochester furnished the following article, which we copy from the *Buffalo Express:*

"The deceased was the only son of the late Hon. Geo. R. Babcock, and was born in this city in 1840. He was carefully educated, and after spending two or three years at Yale College, left before graduating. In 1860 he began the study of medicine with Dr. Rochester, and at once evinced a special aptitude for the profession. He became so proficient that in 1862, before graduating, he passed the rigid examination of the U. S. Naval Board in Philadelphia and was appointed Assistant Surgeon in the navy. One year later, in 1863, he received his diploma from the Buffalo Medical College. The great rebellion was then in progress, and the young doctor served his country through it with fidelity and devotion. He retained his position in the navy until 1869, when he resigned to accept the position of surgeon in the line of steamers running between San Francisco and Yokohama. After one year's service in this capacity he resigned and settled down to private practice at Oakland, Cal. He soon established an enviable reputation and acquired a prominent position among the medical fraternity of that locality. He was elected President of the Alameda County Medical Society, and at the time of his death was President of the Board of Medical Examiners of the State of California. About seven months ago his health began to fail, and it was supposed he was suffering from disease contracted by exposure in the East Indies, and more particularly from a shipwreck in the Indian Ocean. At that time with many other United States naval officers and seamen he was exposed on a raft to a driving sea for fifty-eight hours without food or drink. His health rapidly declining, he determined to come to Buffalo to consult his old friend and preceptor in whom he had the most implicit confidence. All alone he made the long journey from Oakland to Buffalo, sustained by the indomitable pluck and spirit which had ever charcterized him. He was completely exhausted on his arrival, but the day after rallied sufficiently to undergo an operation for his relief. Ten days afterwards the operation was repeated. On both occasions it resulted in great temporary benefit to him. He was unable, however, to fully recover from his long and prostrating illness, and he finally passed away calmly and peacefully, sustained to the last by the true spirit of Christian hope and resignation. Dr. Babcock was married in 1863 to Sarah H., daughter of Commodore Harwood, U. S. N. His wife survives together with two sons, the youngest seven years of age. He also leaves one sister, Mrs, Emily B. Allward, of Auburn

As a physician the deceased was thorough and complete in all his acquirements, remarkably studious and energetic, and possessed of an excellent judgment. He was a deep thinker as well as a fluent writer, and contributed largely to the medical journals of his adopted State. In such high esteem was he held by his associates in the State Medical Board, that they refused to accept his resignation when he tendered it on account of ill health. He was given an unlimited leave of absence in the hope that he would recover from his illness and resume his valuable labors in the Board. In social life he was kind hearted and generous to a fault, and his name and memory will long remain green in the hearts of those who knew him.

